# On Intermittent Pneumonia

**Published:** 1853-11

**Authors:** 


*On Intermittent Pneumonia.* By Dr. CONSTANT.

Dr. Constant, practising in one of the marshy districts of the department of the Lot, draws attention to the signs which distinguish what he terms intermittent pneumonia, as when they are overlooked the disease proves rapidly fatal.

1. The initial shivering is usually more intense and prolonged than in ordinary pneumonia. 2. The pleuritic pain is felt early, and always in front of the chest, although the pulmonary congestion is almost always localized posteriorly. It is much more amenable to blisters than to leeches. 3. Violent cephalalgia is one of the earliest symptoms, being either frontal or sincipital, and it is often accompanied by severe lumbar pain, which observes the same stage of increase and decrease as itself. 4. The shivering is followed by intense heat, which after several hours gives place to abundant sweating. 5. The pulse, during the paroxysm, in place of being full, strong, and vibrating, as in ordinary pneumonia, is rapid, soft, undulant, and compressible. 6. There is never any purulent expectoration, these pneumonias never proceeding beyond the second state—i. e. red hepatization, the pulmonary engorgement being rather a sanguineous congestion than inflammation. 7. Auscultation and percussion are of the highest value, often revealing the disease when unsuspected. A distinctive feature is the rapid passage from the first to the second stage of the disease, so that 8 or 12 hours after auscultation had revealed only a slight circumscribed *rale*, a whole side will be found hepatized. Under the influence of large doses of quinine this rapidly disappears, giving way to returning subscrepitant *rale* during the remission of the fever, but returning again during the paroxysm if this have not been cut short. 8. The crepitant *rale* of the first stage is almost always moist, the parchment-crackling *rale* only having been heard for a short period, two or three times in more than 60 cases It invades large surfaces rapidly, being heard posteriorly, sometimes rapidly, but never in front.. 9. This form of pneumonia especially affects the posterior part of the lower lobes. 10. It especially appears in summer and autumn, while ordinary pneumonia prevails in spring and winter. 11. It attacks all ages indiscriminately, except early infancy. 12. The blood which flows from a vein is often below the normal temperature, very black, and deficient in plasticity. After rest, its surface acquires a bluish color, especially if the patient is taking quinine. The clot is slow in formation, and soft. The buffy coat is absent, or very thin, and inclines to a bluish color. This condition of the blood, conjoined with the soft pulse and rapid hepatization, constitutes the chief distinctive sign of the affection.

In this district, during winter, purely inflammatory pneumonia is met with; but in proportion to the high temperature and the production of malarial emanations, this inflammatory element is replaced by the paludal one. There are indeed three forms met with :—1. Simple pneumonia; 2. Spring inflammatory pneumonia, complicated with the intermittent paroxysm; 3. Summer and autumn intermittent pneumonia. The first requires bleeding and antimony; the second, antiphlogistic treatment with quinine, given either simultaneously or subsequently; and the third, quinine in combination with external revulsives. These forms may still undergo further admixture, accordingly as the inflammatory or paludal element prevails, requiring appropriate modification in the treatment.

